# 非小细胞肺癌分子靶向EGFR-TKI治疗敏感性与治疗耐受性预测筛选的分子显像研究现状及进展

**DOI:** 10.3779/j.issn.1009-3419.2017.12.10

**Published:** 2017-12-20

**Authors:** 东 戴, 文贵 徐, 琦 王, 小凤 李, 研佳 朱

**Affiliations:** 300060 天津，天津医科大学肿瘤医院分子影像及核医学诊疗科，国家肿瘤临床医学研究中心，天津市肿瘤防治重点实验室 Department of Molecular Imaging and Nuclear Medicine, Tianjin Medical University Cancer Institute and Hospital, National Clinical Research Center for Cancer, Tianjin Key Laboratory of Cancer Prevention and Therapy, Tianjin 300060, China

**Keywords:** 肺肿瘤, EGFR, 靶向治疗, PET/CT, 分子显像, Lung neoplasms, EGFR, Targeted therapy, PET/CT, Molecular imaging

## Abstract

肺癌80%以上为非小细胞肺癌（non-small cell lung cancer, NSCLC），表皮生长因子受体（epidermal growth factor receptor, EGFR）介导的信号通路与NSCLC发生发展密切相关。针对EGFR的小分子EGFR赖氨酸激酶抑制剂（EGFR-tyrosine kinase inhibitor, EGFR-TKI）被应用于NSCLC的临床治疗，正电子发射计算机断层显像（positron emission tomography/computed tomgraphy, PET/CT）能够无创地对NSCLC患者全身EGFR表达及突变状况进行连续动态监测。^18^F-FDG PET/CT显像对于*EGFR*活化突变、EGFR-TKI治疗疗效具有预测价值，并且能够在体直接观察到药物与全身肿瘤病灶EGFR靶向结合的具体情况，通过治疗前后的PET-CT显像，实现治疗前高敏人群筛选和治疗全过程的动态监测、治疗策略指导，对实现NSCLC的EGFR-TKI精准治疗至关重要。

## Abstract (en)

More than 80% of lung cancer is non-small cell lung cancer (NSCLC), and the epidermal growth factor receptor (EGFR)-mediated signaling pathway is closely related to the occurrence and development of NSCLC. Small molecule EGFR-tyrosine kinase inhibitors (EGFR-TKI) targeting EGFR have been used in the clinical treatment of NSCLC, and positron emission tomography/computed tomgraphy (PET/CT) imaging can noninvasively monitor the expression and mutation status of EGFR in patients with NSCLC. ^18^F-FDG PET/CT imaging has predictive value for the activation of *EGFR* mutation and EGFR-TKI treatment efficacy, and *in vivo* can be directly observed drugs and systemic tumor targeting EGFR combined with the specific circumstances, by PET/CT imaging before and after treatment, to achieve dynamic monitoring, guide the therapy before treatment and treatment of sensitive population screening process, to achieve NSCLC EGFR-TKI precise treatment is essential.

据统计在2012年，肺癌位居众多恶性肿瘤相关死亡率的首位，约占恶性肿瘤总体死亡率的1/5^[1]^。其中，约80%-85%的肺癌是非小细胞肺癌（non-small cell lung cancer, NSCLC），其中最常见的为鳞癌、腺癌和大细胞肺癌三种病理亚型^[2]^。临床上大部分NSCLC确诊时已处于中晚期，患者5年总体生存率很低，早期精确诊断和治疗至关重要。随着肿瘤分子靶向治疗的不断成熟，现已发现表皮生长因子受体（epidermal growth factor receptor, EGFR）介导的多条信号通路与NSCLC发生发展密切相关，约40%-80%的肺癌患者高比例表达EGFR。

针对EGFR的单抗隆抗体及小分子的EGFR赖氨酸激酶抑制剂（EGFR-tyrosine kinase inhibitor, EGFR-TKI）被应用于NSCLC的治疗，存在某些*EGFR*基因突变（18、19、21密码子突变）的NSCLC患者对于EGFR-TKI高度敏感，治疗有效率高达60%-90%^[3-6]^，而对于野生型EGFR的NSCLC患者，EGFR-TKI治疗有效率低。但EGFR-TKI治疗并不是对于所有发生*EGFR*突变的NSCLC患者均高度有效，也并不是对于所有的EGFR野生型NSCLC患者均无效，而且约90%患者发生了不同程度的EGFR-TKI治疗获得性耐药和抵抗。临床上急需一种无创的能够对于NSCLC患者全身EGFR表达及突变状况进行连续动态监测的检测手段。正电子发射计算机体层成像（positron emission tomography, PET）显像作为一种功能分子显像，能够对靶分子进行在体的全身动态连续的功能分子显像和监测。

## 1 EGFR与NSCLC

EGFR是一种跨膜糖蛋白，属于受体型酪氨酸激酶（receptor tyrosine kinase, RTK）。EGFR内源性酪氨酸蛋白激酶的活化引起下游一系列细胞信号传导分子的级联激活，从而激活多条信号传导通路。EGFR的高比例表达或异常表达，与肿瘤的发生、发展密切相关，成为众多类型肿瘤研究领域中的热点靶分子。EGFR介导的细胞信号传导通路包括以下3条：Rat sarcoma（RAS）/rapidly accelerated fibrosarcoma（RAF）/mitogen-activated protein kinase（MAPK）信号传导通路、phosphatidylinositol-3-kinase（PI3K）/protein kinase B（AKT）信号传导通路和Janus kinase（JAK）/signal transducers and activators of transcription（STAT）信号传导通路。最先用于NSCLC分子靶向临床治疗实践的抗体或者小分子抑制剂药物针对的靶点为EGFR。研究表明，EGFR蛋白在NSCLC患者中高水平表达，尤其是在肺鳞癌患者中，约90%以上的患者过表达EGFR蛋白，而30%-65%的肺腺癌患者高水平表达EGFR蛋白^[7]^。

## 2 NSCLC的EGFR分子靶向治疗及耐药机制

近年来，分子靶向治疗成为多种肿瘤的一种重要治疗手段，显著改善了患者的生活质量，生存期明显延长^[8]^。针对EGFR的分子靶向治疗及耐药机制研究成为NSCLC分子靶向治疗领域的重要方面和研究热点^[9-12]^。EGFR分子靶向治疗主要包括抗EGFR抗体治疗和小分子EGFR-TKI治疗。EGFR-TKI通过与ATP竞争性结合胞内TK位点，从而从EGFR水平阻断其介导的多种细胞信号传导通路的级联激活和活化。

### 2.1 第一代EGFR-TKI

吉非替尼（gefitinib）也称易瑞莎，属于可逆性EGFR-TKI，是第一个被食品药品监督管理局（Food and Drug Administration, FDA）批准用于治疗晚期NSCLC的EGFR-TKI。2009年被批准用于具有EGFR活化突变的NSCLC患者的一线治疗^[13, 14]^。与传统化疗相比，能够显著提高NSCLC患者的无进展生存期^[13-15]^。厄洛替尼（erlotinib）也称特罗凯，与吉非替尼结构相类似。在无进展生存期方面，厄洛替尼治疗组相对于传统化学治疗组均具有明显优势^[16, 17]^。后期开展了其与吉非替尼用于治疗*EGFR*突变NSCLC患者的平行对比研究^[18]^。拉帕替尼（lapatinib）用于NSCLC治疗并不理想，只有25%的患者在治疗后24周表现为病情稳定。埃克替尼（icotinib）是国内一种口服的可逆EGFR-TKI药物，埃克替尼治疗组的中位PFS并不差于吉非替尼治疗组^[19]^。这对于我国自主研发EGFR-TKI药物具有重要意义。此外，有多种小分子新型EGFR-TKI药物被陆续研发和接受临床测试。

### 2.2 第二代EGFR-TKI

阿法替尼（afatinib）是一种口服不可逆的EGFR-TKI药物，于2013年被FDA批准用于具有EGFR突变的NSCLC患者的一线治疗^[20]^。与传统化学治疗相比，阿法替尼治疗组的PFS显著优越于化学治疗组，治疗反应率高达56%^[21]^。达克替尼（dacomitinib)是在模拟吉非替尼结构的基础上加入了迈克尔受体^[22]^。研究^[23]^表明，对于具有EGFR L585R/T790M双突变的非吸烟NSCLC患者而言，4个月的PFS率高达96%，1年的PFS率达到77%，中位PFS为17个月，而且74%的患者经过埃克替尼治疗后达到了部分缓解水平。

### 2.3 第三代EGFR-TKI

第三代EGFR-TKI为不可逆的选择性EGFR-TKI药物，致力于克服T790M介导的药物抵抗，已经显示出了令人鼓舞的疗效，同时对*EGFR*敏感突变亦有抑制作用，有望成为一线治疗的选择。主要药物有osimertinib（AZD9291）、rociletinib（CO1686）、olmutinib（HM61713）、EGF816、ASP8273等。

### 2.4 EGFR-TKI耐药机制

具有活化突变*EGFR*的NSCLC患者在经历吉非替尼/厄洛替尼EGFR-TKI治疗后1年左右，绝大部分患者会产生EGFR-TKI治疗耐受和抵抗。其中耐受机制有多种^[24]^，目前比较公认耐受机制包括：（1）*EGFR*第二突变的发生；（2）EGFR替代通路的异常活化；（3）EGFR下游信号传导通路异常；（4）EGFR-TKI介导的细胞凋亡通路受损；（5）组织学转化；（6）ATP结合盒子的泻流；（7）*EML4*-*AL*融合基因及*ALK*第二突变。

## 3 常规PET/CT显像在NSCLC的EGFR-TKI分子靶向治疗中的应用

以PET/CT为代表的功能分子显像利用放射性核素标记的分子探针（最常用的探针：^18^F-FDG）无创性地与胞内靶分子相结合，由于高灵敏度和高分辨率相结合的独特优势，在肿瘤临床实践的众多决策制定阶段具有重要价值^[25]^。

### 3.1 ^18^F-FDG PET/CT显像对于*EGFR*活化突变的预测研究

^18^F-FDG PET/CT显像用于NSCLC患者EGFR的活化突变预测，不同课题组的研究结果不尽相同^[26-29]^。2011年一项回顾性研究认为突变组相对于非突变组SUVmax较低，SUVmax≥5.0可作为*EGFR*突变与否的临界预测值^[26]^。而2014年另一研究发现，无论是SUVmax，还是平均SUV（SUVmean），*EGFR*突变组与未突变组间均没有显著差异^[30]^，建议SUVmean可作为NSCLC患者*KRAS*突变状态的一个独立预测因子。2015年一项*meta*分析^[29]^结果表明无论是EGFR还是KRAS，^18^F-FDG摄取值均不能预测其突变状态，反而是NSCLC病理类型、吸烟史、肿瘤大小等这些临床特征与突变状态具有相关性。近年来，除SUV外，PET/CT可定量的一些指标应用越来越多，如瘦体重标准化摄取值（standard uptake value of lean body mass, SUL）、肿瘤代谢体积（metabolic tumor volume, MTV）以及葡萄糖酵解总量（total lesion glycolysis, TLG）等，新的研究可将更多的定量指标纳入，以期能获得有意义的结论。

### 3.2 ^18^F-FDG PET/CT显像对于NSCLC的EGFR-TKI治疗疗效的预测研究

PET/CT显像对于NSCLC的早期监测意义重大，从而能够用于指导后续的治疗以及生存预后。研究结果^[31]^表明，^18^F-FDG PET/CT在NSCLC患者的EGFR-TKI治疗过程中具有重要的疗效监测和生存预后价值，治疗前后代谢改变与日后根据实体瘤反应评价标准（Response Evaluation Criteria in Solid Tumors Criteria, RECIST）获得的临床和CT影像改变相一致^[32-34]^。在生存预后方面，PET/CT监测到的代谢反应与否与预后密切相关，包括无进展生存期（progression-free survival, PFS）^[32, 34]^和总生存期（overall survival, OS）^[32-34]^，即代谢反应的存在预示着良好的生存预后。

## 4 EGFR-TKI靶向的PET-CT显像研究与进展

NSCLC的EGFR-TKI治疗是一种EGFR特异性的靶向治疗，选用合适的EGFR-TKI药物进行放射性核素标记作为PET-CT显像探针，能够在体直接观察到药物与全身肿瘤病灶EGFR靶向结合的具体情况，并通过治疗前后对比，实现高敏人群筛选和治疗动态监测、治疗策略指导。

### 4.1 EGFR-TKI靶向的^11^C-PD153035 PET-CT显像研究

基于EGFR-TKI的PET/CT分子显像剂，最初研究比较多的是ML01、ML03、ML04、ML06等^[35]^，其中ML01与PD-153035属可逆性的EGFR-TKI，其他为不可逆性。

1999年首次合成了PD153035的前体化合物^[36]^，并通过动物PET显像最早研究了^11^C-PD153035在体的生物学分布：主要聚集在脑、心脏、肝脏、胃肠道和膀胱等主要脏器。国内于金明院士等在^11^C-PD153035 PET/CT显像方面取得了较高成就。其中王卉^[37]^证实^11^C-PD153035可被移植瘤特异性有效摄取，但由于在胃肠道高浓度聚集分布，可能限制了其在胃肠道肿瘤方面的应用。刘宁波等^[38]^发现在人体，^11^C-PD153035主要通过肝肾系统排泄，在肺脏、骨髓和肌肉中非特异性摄取聚焦较少，在肺脏肿瘤显像方面占据较高的信噪比优势，尤其是在NSCLC的EGFR分子靶向显像方面具有良好的应用前景，因为^11^C-PD153035在肿瘤组织的放射性摄取与肿瘤大小及注射剂量不相关，而是与肿瘤细胞表面表达的EGFR蛋白水平密切相关^[39]^。2011年，孟雪等^[40]^研究表明，erlotinib治疗前^11^C-PD153035 PET/CT显像的SUVmax与NSCLC患者的PFS和OS密切相关。所以，^11^C-PD153035 PET/CT显像可用于EGFR-TKI治疗敏感人群的治疗前筛选，而对于NSCLC的EGFR-TKI治疗疗效检测具有一定的限制性。

### 4.2 EGFR-TKI靶向的^11^C-erlotinib PET/CT显像研究

2009年，丹麦学者首次对erlotinib前体进行^11^C标记获得了^11^C-erlotinib，并进行了小动物PET/CT显像^[41]^。Memon研究结果^[41]^表明，^11^C-erlotinib能够在erlotinib高度敏感的HCC827荷瘤局部特异性有效聚集，而且聚集持续时间相对较长，提示^11^C-erlotinib能够在体识别对于erlotinib敏感的肿瘤组织。

来自于荷兰学者们再次证实NSCLC病灶组织能够有效摄取^11^C-erlotinb聚集并在PET/CT上得以显像，而且通过定量分析，证明肿瘤组织^11^C-erlotinb摄取量与*EGFR*突变状态密切相关，^11^C-erlotinb在具有*EGFR*活化突变的NSCLC组织局部能够高比例特异性聚集^[42, 43]^。另外两项研究^[44, 45]^结果亦表明：^11^C-erlotinb PET-CT显现能够将EGFR-TKI治疗高度敏感的肿瘤组织与EGFR-TKI治疗不敏感或抵抗的肿瘤组织区分开来，^11^C-erlotinb主要在EGFR-TKI治疗高度敏感的肿瘤组织中高比例特异性聚集。

Slobbe课题组还进行了第二代EGFR-TKI标记的荷瘤裸鼠的PET/CT显像研究（^18^F-afatnib），并且与^11^C-erlotinb的PET/CT显像进行了对比分析^[46]^。研究结果表明，无论是^11^C-erlotinb还是^18^F-afatnib均能有效识别EGFR-TKI敏感移植瘤，在HCC827荷瘤局部高比例特异性聚集，而在EGFR-TKI非敏感或抵抗的A549和H1975荷瘤组织局部不能有效聚集。

综上所述，NSCLC的EGFR-TKI治疗作为一种EGFR特异性的靶向治疗，能够明显改善患者生活质量，PET/CT显像能够无创地对NSCLC患者全身EGFR表达及突变状况进行连续动态监测，能够在体直接观察到药物与全身肿瘤病灶EGFR靶向结合的具体情况，通过治疗前后的PET-CT显像，实现治疗前高敏人群筛选和治疗全过程的动态监测、治疗策略指导，在NSCLC患者的EGFR-TKI治疗敏感性与治疗耐受性预测筛选中发挥着不可替代的作用。

## References

[b1] 1World Health Organization. GLOBOCAN 2012: Estimated cancer incidence, mortality and prevalence worlwide in 2012. 2012. Available at: http: //globocan. iarc. fr/Pages/fact_sheets_cancer. aspx. (Accessed on March 2015).

[2] Ettinger DS, Akerley W, Bepler G (2010). Non-small cell lung cancer. J Natl Compr Canc Netw.

[3] Mitsudomi T, Kosaka T, Endoh H (2005). mutations of the epidermal growth factor receptor gene predict prolonged survival after gefitinib treatment in patients with non-small-cell lung cancer with postoperative recurrence. J Clin Oncol.

[4] Mu XL, Li LY, Zhang XT (2005). Gefitinib-sensitive mutations of the epidermal growth factor receptor tyrosine kinase domain in chinese patients with non-small cell lung cancer. Clin Cancer Res.

[5] Han SW, Kim TY, Hwang PG (2005). Predictive and prognostic impact of epidermal growth factor receptor mutation in non-small-cell lung cancer patients treated with gefitinib. J Clin Oncol.

[6] Janne PA, Johnson BE (2006). Effect of epidermal growth factor receptor tyrosine kinase domain mutations on the outcome of patients with non-small cell lung cancer treated with epidermal growth factor receptor tyrosine kinase inhibitors. Clin Cancer Res.

[7] Sequist LV, Lynch TJ (2008). EGFR tyrosine kinase inhibitors in lung cancer: an evolving story. Annu Rev Med.

[b8] 8Minguet J, Smith KH, Bramlage P. Targeted therapies for treatment of non-small cell lung cancer -recent advances and future perspectives. Int J Cancer 2015, [Epub ahead of print].

[9] Steuer CE, Ramalingam SS (2015). Targeting EGFR in lung cancer: Lessons learned and future perspectives. Mol Aspects Med.

[10] Russo A, Franchina T, Ricciardi GR (2015). A decade of EGFR inhibition in *EGFR*-mutated non small cell lung cancer (NSCLC): Old successes and future perspectives. Oncotarget.

[11] Asami K, Atagi S (2014). Epidermal growth factor receptor tyrosine kinase inhibitors for non-small cell lung cancer. World J Clin Oncol.

[12] Lee CC, Shiao HY, Wang WC (2014). Small-molecule EGFR tyrosine kinase inhibitors for the treatment of cancer. Expert Opin Investig Drugs.

[13] Maemondo M, Inoue A, Kobayashi K (2010). Gefitinib or chemotherapy for non-small-cell lung cancer with mutated *EGFR*. N Engl J Med.

[14] Mitsudomi T, Morita S, Yatabe Y (2010). Gefitinib versus cisplatin plus docetaxel in patients with non-small-cell lung cancer harbouring mutations of the epidermal growth factor receptor (WJTOG3405): an open label, randomised phase 3 trial. Lancet Oncol.

[15] Han JY, Park K, Kim SW (2012). First-SIGNAL: first-line single-agent iressa versus gemcitabine and cisplatin trial in never-smokers with adenocarcinoma of the lung. J Clin Oncol.

[16] Zhou C, Wu YL, Chen G (2011). Erlotinib versus chemotherapy as first line treatment for patients with advanced *EGFR* mutation-positive non-small-cell lung cancer (OPTIMAL, CTONG-0802): a multicentre, open-label, randomised, phase 3 study. Lancet Oncol.

[17] Rosell R, Carcereny E, Gervais R (2012). Erlotinib versus standard chemotherapy as first-line treatment for European patients with advanced *EGFR* mutation positive non-small-cell lung cancer (EURTAC): a multicentre, open-label, randomised phase 3 trial. Lancet Oncol.

[b18] 18Erlotinib and gefitinib offer similar benefit in *EGFR*-mutated non-small cell lung cancer. Available from: http: //www. ascopost. com/ViewNews. aspx?nid=15056 [Last accessed 22 April 2014].

[19] Shi Y, Zhang L, Liu X (2013). Icotinib versus gefitinib in previously treated advanced non-small-cell lung cancer (ICOGEN): a randomised, double-blind phase 3 non-inferiority trial. Lancet Oncol.

[20] Passaro A, Gori B, de Marinis F (2013). Afatinib as first-line treatment for patients with advanced non-small-cell lung cancer harboring *EGFR* mutations: focus on LUX-Lung 3 and LUX-Lung 6 phase Ⅲ trials. J Thorac Dis.

[21] Sequist LV, Yang JC, Yamamoto N (2013). Phase Ⅲ study of afatinib or cisplatin plus pemetrexed in patients with metastatic lung adenocarcinoma with *EGFR* mutations. J Clin Oncol.

[22] Brzezniak C, Carter CA, Giaccone G (2013). Dacomitinib, a new therapy for the treatment of non-small cell lung cancer. Expert Opin Pharmacother.

[23] Kris MG, Mok T, Ou S-HI (2012). First-line dacomitinib (PF-00299804), an irreversible pan-HER tyrosine kinase inhibitor, for patients with *EGFR* mutant lung cancers. J Clin Oncol.

[24] Huang L, Fu L (2015). Mechanisms of resistance to EGFR tyrosine kinase inhibitors. Acta Pharm Sin B.

[25] Sharma P, Singh H, Basu S (2013). Positron emission tomography-computed tomography in the management of lung cancer: An update. South Asian J Cancer.

[26] Mak RH, Digumarthy SR, Muzikansky A (2011). Role of ^18^F-fluorodeoxyglucose positron emission tomography in predicting epidermal growth factor receptor mutations in non-small cell lung cancer. Oncologist.

[27] Huang CT, Yen RF, Cheng MF (2010). Correlation of F-18 fluorodeoxyglucose positron emission tomography maximal standardized uptake value and *EGFR* mutations in advanced lung adenocarcinoma. Med Oncol.

[28] Na Ⅱ, Byun BH, Kim KM (2010). ^18^F-FDG uptake and *EGFR* mutations in patients with non-small cell lung cancer: a single-institution retrospective analysis. Lung Cancer.

[29] Lee SM, Bae SK, Jung SJ (2015). FDG uptake in non-small cell lung cancer is not an independent predictor of *EGFR* or *KRAS* mutation status: a retrospective analysis of 206 patients. Clin Nucl Med.

[30] Caicedo C, Garcia-Velloso MJ, Lozano MD (2014). Role of[^18^F]FDG PET in prediction of *KRAS* and *EGFR* mutation status in patients with advanced non-small-cell lung cancer. Eur J Nucl Med Mol Imaging.

[31] van Gool MH, Aukema TS, Hartemink KJ (2014). FDG-PET/CT response evaluation during EGFR-TKI treatment in patients with NSCLC. World J Radiol.

[32] Mileshkin L, Hicks RJ, Hughes BG (2011). Changes in ^18^F-fluorodeoxyglucose and ^18^F-fluorodeoxythymidine positron emission tomography imaging in patients with non-small cell lung cancer treated with erlotinib. Clin Cancer Res.

[33] O'Brien ME, Myerson JS, Coward JI (2012). A phase Ⅱ study of ^18^F-fuorodeoxyglucose PET-CT in non-small cell lung cancer patients receiving erlotinib (Tarceva); objective and symptomatic responses at 6 and 12 weeks. Eur J Cancer.

[34] Takahashi R, Hirata H, Tachibana I (2012). Early[^18^F]fuorodeoxyglucose positron emission tomography at two days of geftinib treatment predicts clinical outcome in patients with adenocarcinoma of the lung. Clin Cancer Res.

[35] Mishani E, Hagooly A (2009). Strategies for molecular imaging of epidermal growth factor receptor tyrosine kinase in cancer. J Nucl Med.

[36] Fredriksson A, Johnstrom P, Thorell JO (1999). *In vivo* evaluation of the biodistribution of ^11^C-labeled PD153035 in rats without and with neuroblastoma implants. Life Sci.

[37] Wang H, Yu JM, Yang GR (2007). Further characterization of the epidermal growth factor receptor ligand ^11^C-PD153035. Chin Med J (Engl).

[38] Liu NB, Li MH, Li XY (2009). PET-based biodistribution and radiation dosimetry of epidermal growth factor receptor-selective tracer ^11^C-PD153035 in humans. J Nucl Med.

[39] Wang H, Yu JM, Yang GR (2007). Assessment of ^11^C-labeled 4-N-(3-bromoanilino)-6, 7-dimethoxyquinazoline as a positron emission tomography agent to monitor epidermal growth factor receptor expression. Cancer Sci.

[40] Meng X, Loo BW Jr, Ma L (2011). Molecular imaging with ^11^C-PD153035 PET/CT predicts survival in non-small cell lung cancer treated with EGFR-TKI: a pilot study. J Nucl Med.

[41] Memon AA, Jakobsen S, Dagnaes-Hansen F (2009). Positron emission tomography (PET) imaging with[^11^C]-labeled erlotinib: a micro-PET study on mice with lung tumor xenografts. Cancer Res.

[42] Bahce I, Smit EF, Lubberink M (2013). Development of[(11)C]erlotinib positron emission tomography for *in vivo* evaluation of EGF receptor mutational status. Clin Cancer Res.

[43] Bahce I, Yaqub M, Errami H (2016). Effects of erlotinib therapy on[(11)C]erlotinib uptake in *EGFR* mutated, advanced NSCLC. EJNMMI Res.

[44] Abourbeh G, Itamar B1, Salnikov O (2015). Identifying erlotinib-sensitive non-small cell lung carcinoma tumors in mice using[(11)C]erlotinib PET. EJNMMI Res.

[45] Petrulli JR, Sullivan JM, Zheng MQ (2013). Quantitative analysis of[^11^C]-erlotinib PET demonstrates specific binding for activating mutations of the EGFR kinase domain. Neoplasia.

[46] Slobbe P, Windhorst AD, Stigter-van Walsum M (2015). A comparative PET imaging study with the reversible and irreversible EGFR tyrosine kinase inhibitors[(11)C]erlotinib and[(18)F]afatinib in lung cancer-bearing mice. EJNMMI Res.

