#  

**DOI:** 10.3779/j.issn.1009-3419.2020.01.11

**Published:** 2020-01-20

本刊2017年第20卷第12期刊登的题为“非小细胞肺癌分子靶向EGFR-TKI治疗敏感性与治疗耐受性预测筛选的分子显像研究现状进展”[戴东, 徐文贵, 王琦, 等.非小细胞肺癌分子靶向EGFR-TKI治疗敏感性与治疗耐受性预测筛选的分子显像研究现状及进展.中国肺癌杂志, 2017, 20(12): 852-856.]一文中：第853页，2.2部分中最后一句话“74%的患者经过埃克替尼治疗后达到了部分缓解水平”应改为“74%的患者经过达克替尼治疗后达到了部分缓解水平”。特此更正。对此深表歉意。

Erratum: Current Status and Progress in Molecular Imaging of Non-small Cell Lung Cancer for Molecular Targeted EGFR-TKI Treatment Sensitivity and Treatment Tolerance Prediction

Dai D, Xu WG, Wang Q, *et al*.

Zhongguo Fei Ai Za Zhi, 2017, 20(12): 852-856.

In the version of this article initially published, error appeared on page 853. The last sentence in part 2.2 should be “74%的患者经过达克替尼治疗后达到了部分缓解水平”.

